# Continuous Flow Methylene Blue Active Substances Method for the Determination of Anionic Surfactants in River Water and Biodegradation Test Samples

**DOI:** 10.1007/s11743-013-1469-x

**Published:** 2013-04-09

**Authors:** Bogdan Wyrwas, Agnieszka Zgoła-Grześkowiak

**Affiliations:** Institute of Chemistry and Technical Electrochemistry, Poznan University of Technology, ul. Piotrowo 3, 60-965 Poznań, Poland

**Keywords:** Anionic surfactant, Dedecylbenzene sulfonate, Determination, Methylene blue reagent, Continuous flow analysis

## Abstract

Anionic surfactants are commonly determined with the use of the methylene blue active substances (MBAS) standard method, which is time-consuming and labor-intensive. Therefore, new methods for determination of anionic surfactants are needed. In this study, the standard MBAS method for determination of anionic surfactants was modified and adjusted to work in a continuous flow system combined with spectrophotometric measurement. The developed method was found to be satisfactory in terms of sensitivity and precision, with a short time of analysis. The quantification limit for anionic surfactants was at 16 μg L^−1^, with a relative standard deviation of 1.3 % for a model sample and 3.8 % for a river water sample. The results obtained for environmental samples were comparable to those obtained by using the standard MBAS method; however, the developed continuous flow method is faster, more sensitive and consumes smaller doses of chemical reagents.

## Introduction

Surfactants are among the main group of environmental contaminants due to their increasing usage and potential toxicity. Anionic surfactants (AS) are found commonly in products of everyday use, such as detergents or washing agents, and linear alkylbenzenesulfonates are presently the most popular synthetic AS. After entering wastewater treatment plants, such compounds are partially degraded in aerobic conditions and partially adsorbed onto the activated sludge. Ultimately, they enter water or soil and act as a major factor influencing the natural environment. Therefore, controlling the surfactant content in detergents and surface water samples is necessary. Fast, cheap and relatively simple methods are of highest priority for monitoring purposes.

Several methods of determining anionic surfactants exist: titration [1, 2], voltamperometry [3], spectrophotometry [1, 3–6], sensor-based methods [7], flow injection analysis [8–10] or chromatography [11–14]. The use of high performance liquid chromatography allows for both qualitative and quantitative analysis of individual surfactants present in mixtures. Depending on the surfactant, such analysis may be carried out with the use of different separation methods, such as reverse-phase or ion-exchange chromatography [11–14]. Chromatography-based methods of determining anionic surfactants are more sensitive and precise compared to standard methods. However, they are not comprehensive enough for quantitative determination of anionic surfactants during a single analysis due to the large variety of chemical structures in environmental samples. Their usage for determination of modern synthetic surfactants may also be challenging, as surfactants are often mixtures of homologues that are difficult to separate.

Growing ecological awareness, enhanced operation of wastewater treatment plants, and increased effectiveness of washing and cleaning systems have all contributed to a decrease in the surfactant content of surface water, despite recent increases in the production of surfactants. Therefore, the search for new and more sensitive methods for surfactant determination is a challenge for modern analytics.

The methylene blue active substances (MBAS) method is a standard method for determination of anionic surfactants [15]. This method is based on the emergence of ionic pairs, which consist of anionic surfactants and a cationic dye (methylene blue), and their transport from water phase to organic phase (chloroform). The sole dye compounds cannot be transported into the organic phase, hence ionic pairs emerge [15–17]. The analytical procedure of the standard MBAS method is carried out with a triple extraction of the ionic pairs from 100 mL of a previously alkalized sample (15, 10 and 10 mL chloroform) and measuring the absorbance of the extract at *λ* = 650 nm [5, 15]. The MBAS method is useful, cheap and simple; however, the procedures are rather troublesome and time-consuming. Using chloroform in high doses is another flaw, as this compound is toxic and harmful for humans and the environment.

Koga et al. [6] succeeded in simplifying the method and shortening the analysis time by omitting part of the classic procedure. The proposed method needs only a single extraction without any prior alkalization of the sample. The sample volume needed for analysis was cut by half. The amount of chloroform used during the analysis was decreased ten-fold and the time required for analysis six times shorter. None of these changes influenced the precision and effectiveness of the method [6]. This procedure was adapted to continuous flow analysis conditions and several optimization studies were carried out, which improved precision and reduced time of analysis still further.

Further simplification of the MBAS method was proposed by Jurado et al. [5]. In the suggested procedure, 5 mL sample were extracted with 4 mL chloroform in a cuvette for a UV spectrophotometer. The chloroform extract was deposited in the lower part of the cuvette and analyzed without filtration [5].

In the present paper, a continuous flow MBAS procedure is proposed. Up to 100 mL sample can be extracted with 5 mL chloroform. The extract is transferred continuously to a spectrophotometric cuvette for measurement. The final procedure is fast, simple and enables high enrichment of the sample.

## Materials and Methods

### Apparatus and Reagents

#### Spectrophotometer

Spectrophotometric determination of anionic surfactants was carried out at *λ*_max_ = 652 nm with the use of a UV–Vis spectrophotometer (Jasco, Tokyo, Japan) equipped with a flow cuvette SFC-333 (5 mm × 10 mm × 5 mm, 10 mm optic path).

#### Reagents

The anionic surfactant sodium dodecylbenzenesulfonate (SDBS) was purchased from Sigma-Aldrich (St. Louis, MO). The non-ionic surfactant dodecyl alcohol ethoxylate (OXETAL 114) was gained from Zschimmer and Schwarz (Lahnstein, Germany).

The reagents used for analysis included methylene blue from Sigma-Aldrich and chloroform from POCh (Gliwice, Poland). The salts needed for preparation of the mineral medium used in the biodegradation test were from POCh.

Methylene blue solution was prepared in an Erlenmeyer flask (1,000 mL) by dissolving 0.35 g methylene blue in 500 mL redistilled water and adding 6.5 mL concentrated H_2_SO_4_. Afterwards the flask was filled with redistilled water. The solution obtained was purified by triple extraction with chloroform (10 mL) to remove chloroform soluble contaminants. The solution was prepared 24 h before usage.

### Description of the Determination Method

**Fig. 1:**
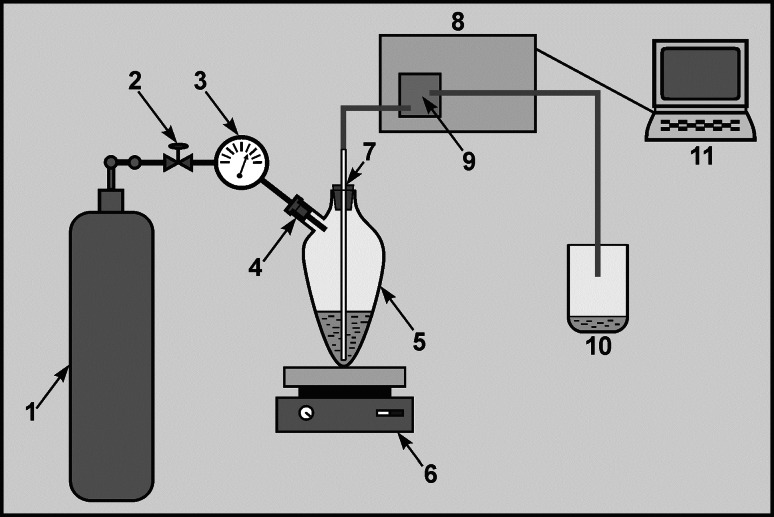
Continuous flow system for determination of anionic surfactants. *1* Nitrogen gas tank, *2* valve, *3* manometer, *4* extraction vessel entry, *5* dosing extraction vessel (100 mL), *6* magnetic stirrer, *7* PTFE tube for sample transport, *8* UV–VIS spectrophotometer, *9* flow cuvette, *10* wastewater, *11* computer

The sample to be analyzed was placed into an extraction vessel (100 mL) along with 5 mL CHCl_3_ and 5 mL acidic methylene blue solution. The whole content was homogenized for 3 min with a magnetic stirrer. The anionic surfactant reacts with the methylene blue cation and enters the organic phase as an ionic pair. After a complete separation of phases (3 min) the nitrogen valve was opened, leading to a rise in pressure above the sample in the tightly closed extraction vessel. The rising pressure pushed the lower, chloroform phase out of the vessel and the chloroform extract was transported continuously to the bottom of the spectrophotometer flow cuvette with the help of a PTFE tube, to reduce the formation of gas bubbles (Fig. 1). The analytical signal was measured at *λ*_max_ = 652 nm against air. After the analysis, the system was cleaned with three portions of methanol (5 mL) and zeroed with chloroform (5 mL).

### Biodegradation Test and the Determination of Residual Surfactants

An aerobic biodegradation test was carried out for 21 days. Determination of SDBS biodegradation efficiency was performed under aerobic conditions, using a mineral medium inoculated with activated sludge, which was collected from the local waste treatment plant in Szamotuły. The collected sludge was purified for 7 days in the continuous flow Husmann apparatus and afterwards used to inoculate static aerobic biodegradation test samples. The initial concentration of SDBS was 10 mg L^−1^. Samples were collected each day for the 1st week, and afterwards at intervals of 2–3 days. Upon conservation with formalin, the collected samples were subjected to anionic surfactants determination using the developed MBAS continuous flow method, as well as the standard MBAS method.

#### Preparation of Mineral Medium

The mineral medium used for the static biodegradation test was composed of four solutions [18]:

- Solution A: 8.5 g KH_2_PO_4_, 21.75 g K_2_HPO_4_, 33.4 g Na_2_HPO_4_⋅2 H_2_O and 1.7 g NH_4_Cl (per 1,000 mL redistilled water). The pH value of the solution should be around 7.2;
- Solution B: 22.5 g MgSO_4_⋅7 H_2_O (per 1,000 mL redistilled water);
- Solution C: 27.5 g CaCl_2_ (per 1,000 mL redistilled water);
- Solution D: 0.25 g FeCl_3_⋅6H_2_O (per 1,000 mL redistilled water).

The amount of each solution used to prepare 1,000 mL of mineral medium: 10 mL solution A; 1 mL each of solutions B, C, D; redistilled water (filled to 1,000 mL).

#### Biodegradation Test

The biodegradation test for an anionic surfactant (SDBS) was carried out in the presence of a non-ionic surfactant—OXETAL 114 (oxyethylated alcohol C12E14). These surfactants were the only sources of carbon and energy. The biodegradation test was performed in a biodegradation dish (15 L) containing: 8 L water, 80 mg SDBS (initial concentration 10 mg L^−1^), 80 mg C12E14 (initial concentration 10 mg L^−1^), mineral medium (80 mL solution A, 8 mL each of solutions B, C, D), 100 mL activated sludge inoculum (3.5 g dry matter L^−1^).

An oxygen pump was used to aerate the biodegradation dish, although only periodically, to reduce the formation of foam during the first stage of the test. The oxygen concentration was kept at 2–3 mg L^−1^ and controlled during every 24 h by a calibrated digital oxygen sensor. Aeration intervals were lengthened for low oxygen levels and shortened for high oxygen levels while periods without aeration were kept constant. Samples were collected with the help of an injector (100 mL), each day for the 1st week, and afterwards at intervals of 2–3 days. In order to avoid the injection of suspensions and sediments, the samples were collected from under the liquid surface after stopping the aeration (~60 min). Upon conservation with formalin (5 mL), the collected samples (300 mL) were subjected to anionic surfactants determination using the developed MBAS continuous flow method.

## Results and Discussion

### Basic Parameters of the Method

**Fig. 2:**
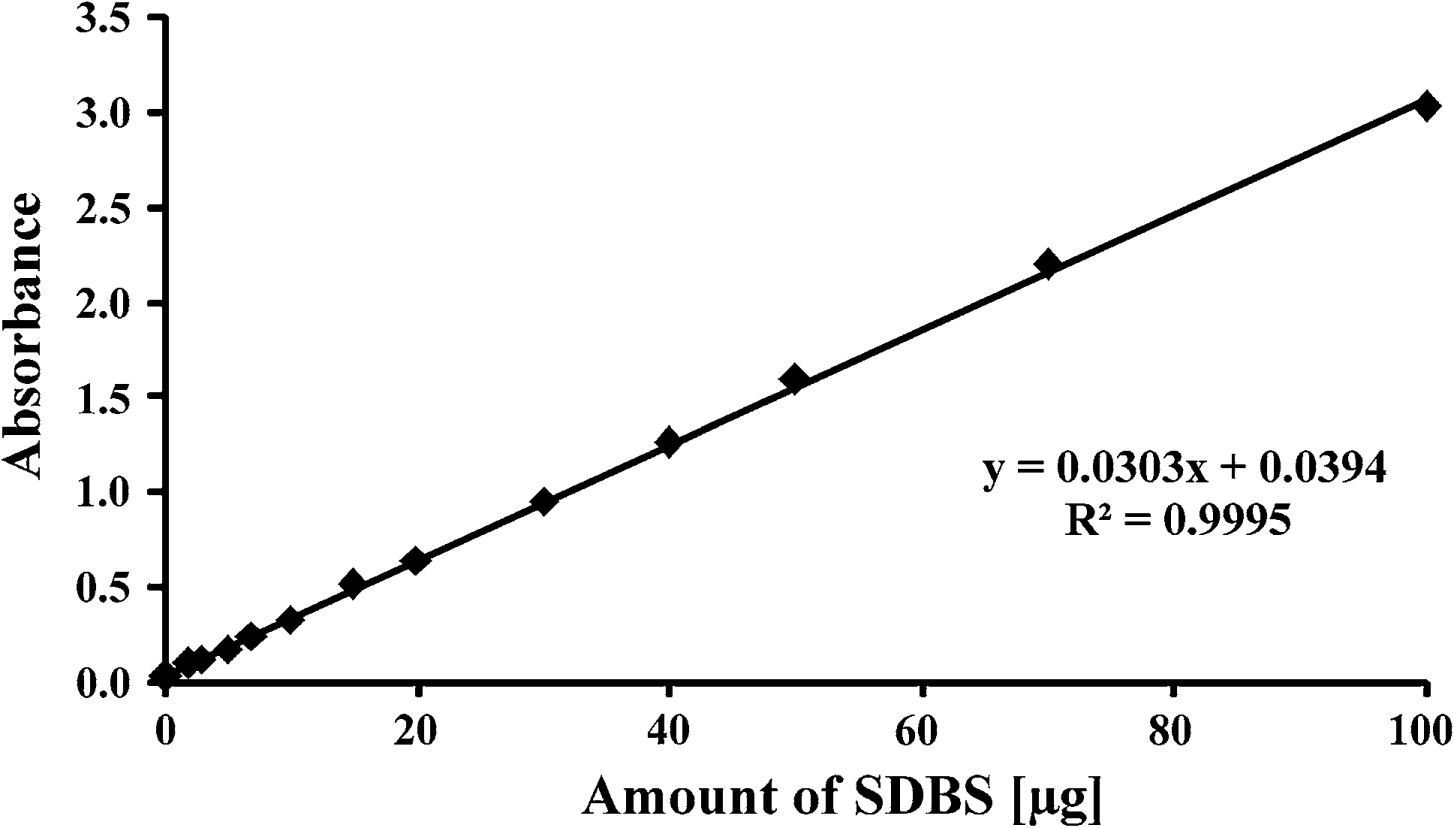
Correlation between measured absorbance value and the amount of sodium dodecylbenzenesulfonate (SDBS) for the developed methylene blue active substances (MBAS) continuous flow method

The analytical signal for the anionic surfactant-methylene blue ion pair was measured at *λ* = 652 nm in a continuous flow system. The correlation between the measured absorbance of the chloroform phase and the amount of surfactant added to the water phase is linear up to 100 μg SDBS (Fig. 2), i.e., to 20 μg mL^−1^ in 5 mL of the chloroform phase. For maximum volume of the water phase equal to 100 mL, the sample is enriched 20 times and concentration can be measured up to 1,000 μg L^−1^ (100 μg in the sample vessel). However, use of lower volumes of water samples enables extension of the working range of the method above this limit, i.e., up to 20,000 μg L^−1^ for 5 mL water sample.

Precision of the method was evaluated for a model sample containing 5 μg of the anionic surfactant SDBS (Table 1). The detection limit (DL) for the method presented was calculated according to Sádecká and Polonský [19]. For the limit of detection, the value (*x*_0_ + 3*σ*)/*b* was used, where *x*_0_ is the mean absorbance of blank samples, *σ* is the standard deviation for blank samples, and *b* is the slope of the calibration line. For the quantitation limit (QL) the value *(x*_0_ + 10*σ*)/*b* was used. The results obtained for consecutive measurements of blank samples (Table 1) and linearity (Fig. 2) enabled calculation of DL at 1.45 μg and QL at 1.62 μg. For a maximum sample volume of 100 mL, DL = 14.5 μg L^−1^ and QL = 16.2 μg L^−1^.

**Table 1:** Precision of measurement for sample containing 5 μg SDBS and fluctuations of blank value

No.	Absorbance for sample containing 5 μg SDBS	Determined SDBS content (μg)	Absorbance for blank	Determined SDBS content (μg)
1	0.1775	4.56	0.0422	0.0924
2	0.1744	4.45	0.0406	0.0396
3	0.1735	4.43	0.0417	0.0759
4	0.1779	4.57	0.0419	0.0825
5	0.1756	4.50	0.0415	0.0693
6	0.1736	4.43	0.0426	0.1056
7	0.1715	4.48	0.0427	0.1089
Mean	0.1749	4.49	0.0419	0.0820
SD	0.0023	0.0582	0.0007	0.0238
RSD (%)	1.31	1.30	1.72	29.0

### Optimization of Analytical Procedure Parameters

#### Optimization of Extraction Time

**Fig. 3:**
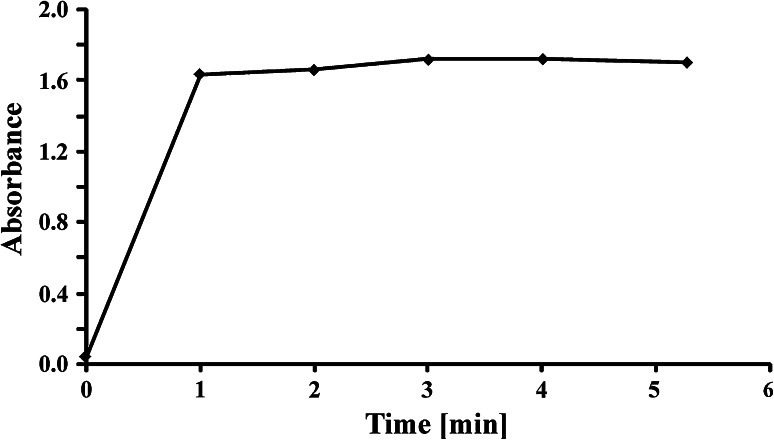
Influence of sample stirring time on the measured absorbance value for 50 μg SDBS

Stirring is a significant element in the whole analytical procedure, allowing transportation of surfactants into the chloroform phase. Extraction time was investigated, ranging between 0 and 5 min, for a model water sample containing 50 μg of a standard (SDBS). On the basis of the obtained results (Fig. 3) it was concluded that similar but not constant absorbance was recorded in the range from 1 to 5 min. As signal obtained for the range from 3 to 5 min (absorbance equal to 1.72) was slightly higher than signal for the range from 1 to 2 min (absorbance equal to 1.65), a stirring time of 3 min was used in further studies.

#### Optimization of Phase Separation Time

The time needed for complete separation of the water and chloroform phases after the stirring process was investigated. The chloroform phase was transferred to the spectrophotometer cuvette directly after stirring was finished and after 1, 3, 5 and 7 min. Absorbance measured during this test was constant. This suggests that a direct measurement of the chloroform phase absorbance for the model samples is possible even right after the stirring stage. However, a 3 min separation time was used in further studies to ensure proper phase separation for environmental samples. This may be a significant step for these samples due to the possible formation of emulsions.

#### Optimization of Amount of Chloroform 

The influence of amount of chloroform (ranging between 1 mL and 10 mL) on the results obtained was investigated in the hope of achieving an improved method sensitivity and reducing chloroform consumption. Measurements were performed with a model sample containing 50 μg SDBS. The absorbance could not be measured using 1 mL and 2 mL chloroform phase. It was possible to use 3 mL chloroform in the analytical procedure; however, it was concluded that using 5 mL chloroform results in improved repeatability. Further increase in chloroform volume had no influence on repeatability of the proposed method. Therefore, 5 mL was taken as the optimal volume.

#### Optimization of the Amount of Methylene Blue Solution

**Fig. 4:**
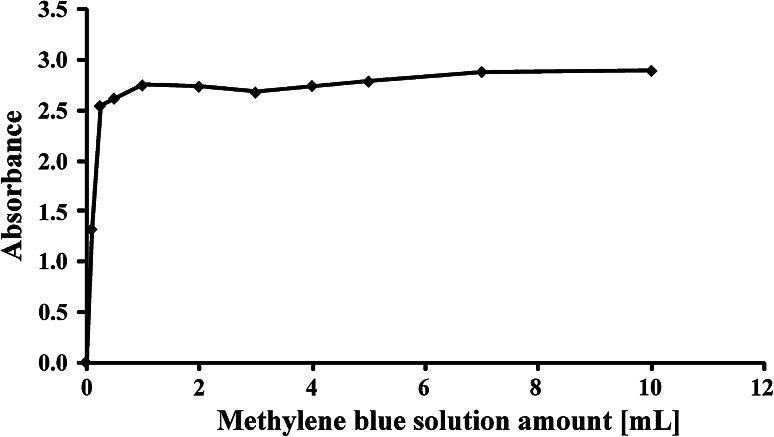
Influence of amount of methylene blue solution on the absorbance obtained for a sample containing 100 μg SDBS

An appropriate volume of methylene blue solution should be used in the analysis to ensure its excess and extraction of the ionic pair formed. The maximum possible amount of SDBS should be used in this test to enable future use of the developed procedure for the entire range of concentrations. Therefore, optimization was carried out for a model sample containing 100 μg SDBS. The investigated methylene blue amount ranged from 0.1 mL to 10 mL. A considerable increase in absorbance was noted up to a volume of 1 mL (Fig. 4). Any further increase in methylene blue volume had no influence on absorption. However, taking into account the potential increase of the sample volume during analysis of environmental samples, it was concluded that using 5 mL methylene blue solution is optimal.

#### Optimization of Sample Volume

The influence on the measured absorbance value of the volume of the water sample containing 50 μg anionic surfactant was investigated. No significant influence was recorded from 5 mL to 100 mL. Therefore, it was concluded that different sample volumes can be used to enhance method range.

### Application of the Developed Continuous Flow Method for Analysis of Environmental Samples

#### Application of the Method for Biodegradation Tests

The developed method for determination of anionic surfactants with a continuous flow detection system was used during a static, aerobic biodegradation test. The biodegradation results obtained were compared with results from the standard MBAS determination method and are presented together in Table 2. The data obtained for the standard MBAS method and the MBAS continuous flow method are very similar and confirm fast biodegradation of SDBS.

**Table 2:** Comparison of sodium dodecylbenzenesulfonate (SDBS) determination results from the biodegradation test samples obtained with the developed MBAS continuous flow method and the standard methylene blue active substances (MBAS) method

Test day	Standard MBAS method			MBAS continuous flow method		
	Sample volume (mL)	Absorbance	Determined SDBS concentration (mg L^−1^)	Sample volume (mL)	Absorbance	Determined SDBS concentration (mg L^−1^)
1	100	2.5496	9.40	5	1.340	8.61
2	100	2.2990	8.48	5	1.206	7.70
3	100	2.0166	7.46	10	2.316	7.51
4	100	1.5305	5.69	15	2.912	6.32
5	100	1.2215	4.56	20	3.026	4.92
6	100	0.1741	0.75	30	1.240	1.31
7	100	0.0744	0.39	40	0.826	0.65
8	100	0.0476	0.29	50	0.840	0.53
10	100	0.0788	0.41	60	0.905	0.48
13	100	0.0471	0.29	70	1.018	0.46
16	100	0.0609	0.34	80	1.009	0.39
21	100	0.0384	0.26	100	0.853	0.27

There was no significant difference between the results obtained with the two methods for higher SDBS concentrations. For lower concentrations, the continuous flow method gave higher results than the standard MBAS method and in a few examples the difference can be considered significant. However, it must be taken into account that the samples from biodegradation tests contain substantial amounts of suspended activated sludge. SDBS can be easily adsorbed onto such sludge, leading to lower results in the standard MBAS method, which discards suspended matter during the filtration step.

#### Determination of Anionic Surfactants in River Water

Results obtained for the determination of anionic surfactants content in river water samples (Warta River, Poznań) carried out using the standard MBAS method and MBAS continuous flow method are shown in Tables 3 and 4.

**Table 3:** SDBS recovery from river water samples (100 mL, Warta river, Poznań)

Sample	Amount of SDBS added (μg)	Absorbance	Determined SDBS content (μg 100 mL^−1^ of sample)	Recovery (%)
1	0	0.1976	5.22	–
2	5	0.3295	9.57	93.7
3	10	0.4718	14.27	93.8
4	15	0.6346	19.64	97.2
5	20	0.7782	24.28	96.7
6	30	1.0389	32.98	93.7
7	40	1.3926	44.95	98.8

**Table 4:** Results of anionic surfactant determination by the standard MBAS method and MBAS continuous flow method for river water samples, calculated as SDBS equivalents (Warta River, Poznań)

Sample	Standard MBAS method			MBAS continuous flow method		
	Sample volume (mL)	Absorbance	Concentration (μg L^−1^)	Sample volume (mL)	Absorbance	Concentration (μg L^−1^)
1	200	0.0273	55.0	100	0.1976	52.2
2	200	0.0250	50.0	100	0.1974	52.1
3	200	0.0200	39.0	100	0.1982	52.4
4	200	0.0249	49.9	100	0.1951	51.3
5	200	0.0242	48.3	100	0.1876	48.9
6	200	0.0244	48.8	100	0.2079	55.6
7	200	0.0253	50.7	100	0.1991	52.7
Mean	–	0.0244	48.8	–	0.1976	52.2
SD	–	0.0022	4.84	–	0.0060	1.98
RSD (%)	–	9.03	9.93	–	3.03	3.80

The content of ionic surfactants in Warta River water was determined to be 5.22 μg per 100 mL. Therefore, 100 mL of sample was spiked with 5–40 μg SDBS, i.e., from 100 to 800 % of the level determined in the river water. The recovery obtained ranged from 94 % to 99 % (Table 3) and was satisfactory for all tested samples.

For environmental surface water samples with a low anionic surfactants content, results obtained using the standard MBAS method (49 μg L^−1^) and the MBAS continuous flow method (52 μg L^−1^) were very similar. However, the precision of the newly developed MBAS method was almost three times higher than the standard MBAS method (Table 4).

### Comparison of the Standard MBAS Method and the Developed MBAS Continuous Flow Method for Anionic Surfactant Detection

Due to the systematic decrease in anionic surfactants content in surface water, the standard MBAS method is slowly becoming insufficient for their efficient determination. An environmental sample with a volume of 300 mL (100 mL is used to saturate the filter) is frequently on the edge of the quantification limit set for the standard MBAS method. The newly developed continuous flow method is significantly improved in terms of analytical signal sensitivity compared to the classic method. Only 100 mL unfiltered surface water is enough to perform the determination. It is also worth noting that the developed continuous flow method for anionic surfactant determination is simpler, faster and much more efficient in terms of chemical reagent consumption. The basic parameters of both methods are compared in Table 5.

**Table 5:** Comparison of the standard MBAS method and the developed continuous flow method

No.	Parameter	Standard MBAS method	Developed MBAS continuous flow method
1.	Molar adsorption coefficient (l mol^−1^ cm^−1^)	4.21 × 10^4^	4.79 × 10^4^
2.	Value of the blank	*A* = 0.015	*A* = 0.0419
3.	Reagent consumption for a single sample		
	Sodium carbonate (g)	0.27	–
	Sodium bicarbonate (g)	0.24	–
	Methylene blue (mg)	3.5	1.75
	Chloroform (mL)	50	5
4.	Extraction	Three doses of chloroform, manual shaking	One dose of chloroform, automatic stirring
5.	Analysis time (min)	30	7
6.	Quantification limit (μg)	6.7	1.6
7.	Precision of the method		
	For 5 μg of SDBS standard RSD (%)	19.9	1.3
	For river water sample containing 50 μg L^−1^ of anionic surfactants RSD (%)	9.9	3.8

## Conclusions

An analytical set was constructed using a modified MBAS method for anionic surfactant determination in a continuous flow system combined with spectrophotometric measurement. Compared to the standard MBAS method, the developed method contributes to decreased analysis time, reduced reagent consumption, and increased sensitivity and precision. The developed method may be used successfully not only for model experiments, but also for the determination of anionic surfactant content in biodegradation test samples. Due to the decrease in quantification limit, the developed continuous flow method may also be used for fast, routine control of anionic surfactant level in unfiltered surface water samples. By using appropriate modified reagents in the developed continuous flow analytical set, it is also possible to determine other surfactant types, such as non-ionic or cationic surfactants. Determination studies carried out for such surfactants using the continuous flow system under the proposed conditions are currently being finalized. Under continuous flow conditions, non-ionic surfactants may be determined by using tetrabromophenolophthalein ethyl ester (TBPE) and cationic surfactants may be determined by using disulfine blue (DBAS). The developed continuous flow analytical set is a universal system that may be applied successfully to the analysis of different surfactants, as well as any other compound that exhibits a specific spectrophotometric signal under the given conditions.
